# Integrating the influence of weather into mechanistic models of butterfly movement

**DOI:** 10.1186/s40462-019-0171-7

**Published:** 2019-09-02

**Authors:** Luke C. Evans, Richard M. Sibly, Pernille Thorbek, Ian Sims, Tom H. Oliver, Richard J. Walters

**Affiliations:** 1 School of Biological Sciences, University of Reading, Whiteknights, PO Box 217, Berkshire, Reading RG6 6AH UK; 2 Syngenta, Jealott’s Hill International Research Centre, Bracknell, Berkshire, RG42 6EY UK; 3 Centre for Environmental and Climate Research, University of Lund, Lund, Sweden; 4 BASF SE, APD/EE, Speyerer Strasse 2, 67117 Limburgerhof, Germany

**Keywords:** Body temperature, Climate warming, Lepidoptera, Motivation, Thermoregulation

## Abstract

**Background:**

Understanding the factors influencing movement is essential to forecasting species persistence in a changing environment. Movement is often studied using mechanistic models, extrapolating short-term observations of individuals to longer-term predictions, but the role of weather variables such as air temperature and solar radiation, key determinants of ectotherm activity, are generally neglected. We aim to show how the effects of weather can be incorporated into individual-based models of butterfly movement thus allowing analysis of their effects.

**Methods:**

We constructed a mechanistic movement model and calibrated it with high precision movement data on a widely studied species of butterfly, the meadow brown (*Maniola jurtina*), collected over a 21-week period at four sites in southern England. Day time temperatures during the study ranged from 14.5 to 31.5 °C and solar radiation from heavy cloud to bright sunshine. The effects of weather are integrated into the individual-based model through weather-dependent scaling of parametric distributions representing key behaviours: the durations of flight and periods of inactivity.

**Results:**

Flight speed was unaffected by weather, time between successive flights increased as solar radiation decreased, and flight duration showed a unimodal response to air temperature that peaked between approximately 23 °C and 26 °C. After validation, the model demonstrated that weather alone can produce a more than two-fold difference in predicted weekly displacement.

**Conclusions:**

Individual Based models provide a useful framework for integrating the effect of weather into movement models. By including weather effects we are able to explain a two-fold difference in movement rate of *M. jurtina* consistent with inter-annual variation in dispersal measured in population studies. Climate change for the studied populations is expected to decrease activity and dispersal rates since these butterflies already operate close to their thermal optimum.

**Electronic supplementary material:**

The online version of this article (10.1186/s40462-019-0171-7) contains supplementary material, which is available to authorized users.

## Background

Understanding individual movement is crucial to species conservation as it directly impacts metapopulation stability and species persistence [1]. In order to predict the consequences of anthropogenic change, it is essential to understand, in detail, the capacity and the motivation for movement of species within complex landscapes [2–4]. Butterflies have served as a model systems to investigate movement processes [5] that determine metapopulation dynamics [6], home-range sizes [7, 8], functional connectivity [9], and minimum area requirements [10], though accurately predicting movement rates remains challenging, since movement is context dependent and driven by multiple environmental factors [11].

The drivers of movement behaviour have been variously investigated and modelled in butterflies. Examples include: responses to boundaries [12–16], habitat-specific movement rates [17, 18], and variation among individuals in motivation to move [19]. Progress in modelling these effects is achieved by incorporating mechanisms underlying the behavioural responses to changing conditions. Rarely though has the effect of weather been included (but see [18]), despite the well-established temperature-dependency of lepidopteran flight behaviour [20–25] and the underlying physics of heat transfer being known in detail for *Colias* butterflies [26]. Therefore, the consequences of weather and climate variability on potential movement rates have yet to be fully addressed.

Recent field studies conducted on a number of different butterfly species confirm that weather is an important factor explaining propensity for emigration [27] and underlying the variation in dispersal rate between years [28, 29]. Specifically, rate of movement is found to increase with both air temperature and sunshine intensity due to their predicted independent effects on body temperature [30]. Environmental variability in propensity to move is shown to contribute to the kurtosis of dispersal kernels in general [31–35]. However, while metabolism is expected to increase with temperature under predicted climate change [36], performance is eventually impaired as species approach their thermal safety margins [37–39], forcing a change in thermoregulatory behaviour that can ultimately limit and reduce movement rates [40, 41]. Understanding of these effects is necessary as species ranges are shifting rapidly in response to changing climates [42, 43], and the rates of range shifts are linked to species mobility [44].

In order to better understand and predict the effects of weather on movement rate in butterflies we investigated the weather-dependence of movement behaviour in the model species *Maniola jurtina* (L. 1758). *M. jurtina* is a common species which exists in networks of local fragmented populations. It is a relatively sedentary species with short mean dispersal distances. The majority of individuals remain in their natal patch [45], a situation typical of butterflies in metapopulations [46] making it ideal to model. Various aspects of the movement behaviour of *M. jurtina* have been empirically investigated, notably changes in movement rates with habitat quality and edge responses [47–51]. Both temperature and solar radiation are known to influence the movement rate of a range of butterfly species, including *M. jurtina* [29], though a basis for including these in predictions of movement is lacking. Here we address this issue by introducing an individual-based model which incorporates weather-dependent changes in duration of flights and inactivity (referred to hereafter as *inter-flight durations*). The model is parameterised with extensive high precision data on both flight tracks and behavioural time budgets collected over the course of three seasons and at four sites which demonstrates the influence of weather on flight and inter-flight durations. Movement models incorporating flight and inter-flight have only recently been developed [19] and we show how the influence of weather can also be included. The model is validated with data collected over 10-min intervals and is then used to explore the consequences of a weather on weekly displacement rates. We conclude by discussing possible consequences of these findings for the responses of *M. jurtina* to climate change.

## Methods

### Study species and sites

The meadow brown (*Maniola jurtina*) is a widespread univoltine butterfly with a flight period that extends across the summer months in the UK from June to September [52]. It is commonly found in a variety of grasslands habitats [45], where the larvae feed mainly on *Poa spp* and the adults nectar on a range of flowering plants [53].

Data on individual flight tracks were collected over 72 days during the summers of 2016 (July–August), 2017 (June–September) and 2018 (June–July), at four sites in the south of England: North farm in Oxfordshire (51°37′N, 1°09′W), Jealott’s Hill farm Berkshire (51°27′N, 0°44′W), the University of Reading (51.4414° N, 0.9418° W), and Sonning farm Berkshire (51°28′N, 0°53′W). Three of the sites were agricultural farms which had implemented agri-environment schemes and consisted of a mixture of arable fields, open meadows, and nectar rich field margins, while the fourth consisted of areas of meadow within the grounds of the Reading University campus.

### Movement & behavioural observations

Three hundred eighty-five (♀181, ♂204) individual butterflies were followed at a distance of approximately three metres continuously for up to 10-min intervals to record both movements and behaviour. These distances allow careful observations of the butterflies without disturbing their behaviour. Flight paths were reconstructed as a series of steps and turns between landings and successive 15 s periods of continuous flight [54]. Positions were initially marked with numbered flags, the precise coordinates for which were subsequently mapped using a high-grade Global Navigation Satellite System receiver accurate to < 30 cm (*Arrow* 200 RTK). The time for which an individual was followed, termed *observation time* was either 10 min or after a set number of flags were laid (20 in 2016 & 2017 and 15 in 2018), whichever event occurred first. Step distances and relative turning angle were calculated based upon the coordinates of the successive flagged positions. During the observations activity was recorded continuously by categorising behaviour into: flying and inter-flight with the timing of behaviour recorded accurately using a bespoke android phone app developed for the project by LE. Any flight and inter-flight durations which were ongoing at the end of the observation were treated as right-censored data in subsequent analyses.

We use two measures of 10-min displacement, which we term *distance rate* and *displacement rate.* Distance rate is here defined as the total flight path distance divided by the observation time; displacement rate (m/s) is the Euclidean distance moved during the observation divided by the observation time.

Dataloggers (HOBO pendant) were used to record solar radiation (lux) at 10 s intervals and air temperature was measured at hourly intervals from meteorological stations within 3 km of each site (Jealotts Hill, Sonning, University of Reading, RAF Benson).

### Statistical analysis

Linear models were used to demonstrate the influence of sex, air temperature, (air temperature)^2^, and solar radiation on the movement variables, though a different procedure was used for incorporating these effects into the individual-based model as it is then desirable to model both the changing mean and variance of flight and inter-flight durations across weather categories (see *Generalising behavioural responses to weather conditions)*. (Air temperature) ^2^ was introduced as covariate after visual inspection of the relationship between air temperature and flight duration. To control for repeated measures from an individual, means of the variables were calculated such that each observation of a movement variable referred to a unique individual. Model diagnostics were used to check the conformation of the data to the assumptions of linear models and minimal transformations were used when residuals were skewed, thus step speeds, displacement rate and distance rates were cube-root transformed, and flight and inter-flight durations which were log transformed. Stepwise AIC was used to drop uninformative covariates. The Wall-Raff rank sum tests of angular distance, which is available through the *circular* package in R [55] was used to test for differences in turning angles between the sexes.

### Generalising behavioural responses to weather conditions

The individual-based model required representative distributions fitted to the flight and inter-flight durations across weather conditions. The data was subdivided to allow for changes in both the means and the variance of the representative distribution across the changing weather conditions. To evaluate the effect of temperature on flight duration distributions, flights were ranked by recorded air temperature and then subdivided to give five categories across the observed range (median values: 16.2 °C, 19.6 °C, 23 °C, 26.4 °C, 29.8 °C). Inter-flight duration distributions were similarly analysed across the range between 10 and 230klx as measured on the dataloggers (i.e. from overcast to full sunshine) using median values: 30.2 k lx, 76 klx, 120 klx, 16.4klx, 22.6klx.

Flight and inter-flight durations were long-tailed, and goodness of fit statistics were used to choose between candidate parametric distributions (log-normal distributions were selected as most appropriate). As flight and inter-flight durations contain right censored observations, distributions were fitted using ‘fitdistcens’ an algorithm available in *fitdistplus* package through R [56] which takes account of censoring and uses maximum likelihood methods to fit distributions to data. Flight duration distributions were then fitted across temperature categories and inter-flight durations distributions across solar intensity categories. This allowed evaluation of the change in the parameters of the log-normal distributions (log μ, σ) across weather conditions. The changes were summarised using a quadratic model which was selected after visual inspection of the change in parameters across weather conditions. This provided an estimate of the shape of flight and inter-flight distributions between the upper and lower bounds of the observed weather conditions. All analysis was carried out in R 3.4.2 (R Core Team, 2018).

### Individual based model

**Fig. 1:**
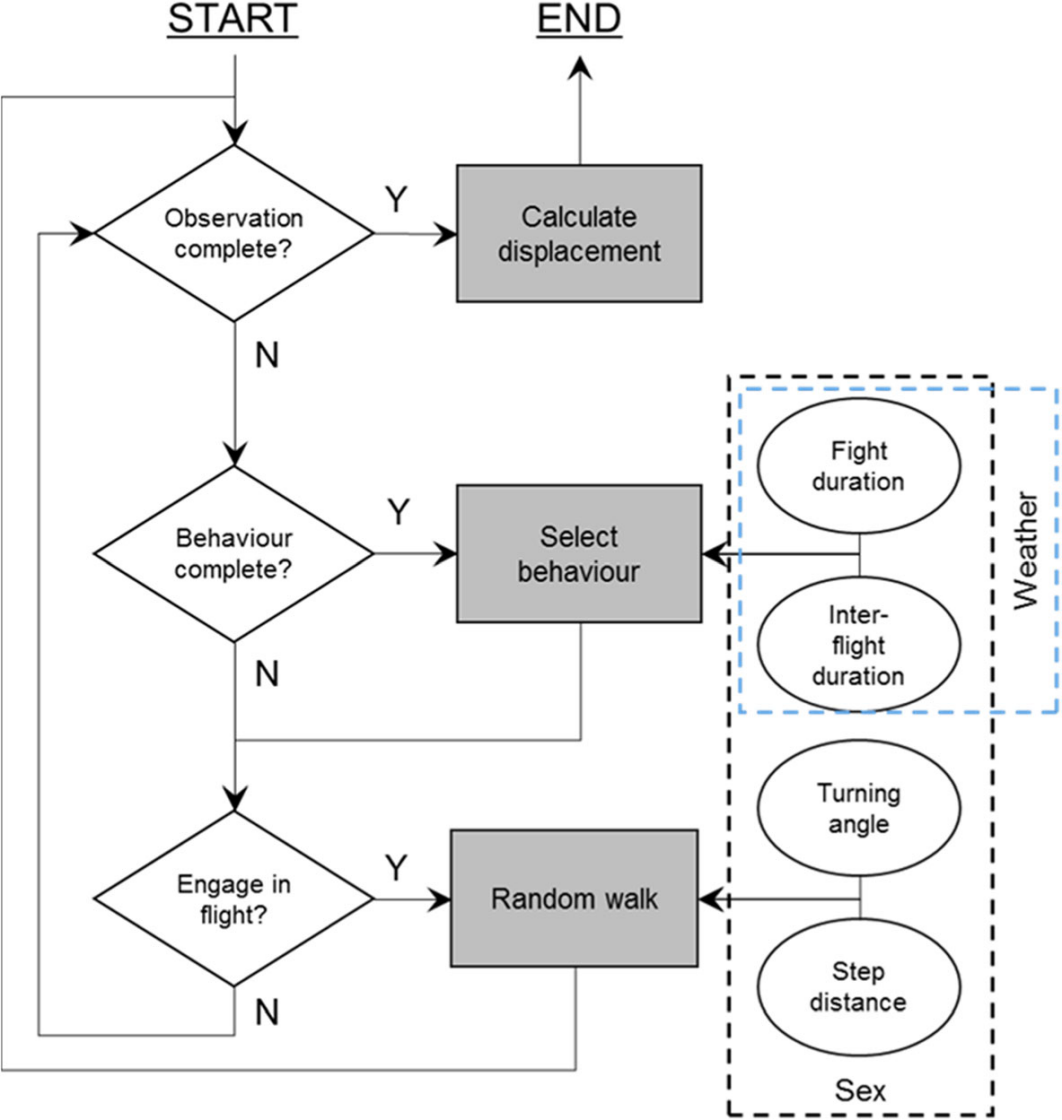
Conceptual model of the IBM. Solid boxes represent model processes, diamonds decision points, and ovals data input to the model. Condition dependence of data input is indicated by dashed boxes. The model runs on a one-second time step

A spatially-explicit individual-based random walk model was developed to evaluate the effect of temperature and solar radiation on the movement rates of *M. jurtina*. The model consists of individuals representing butterflies which move across a grid of habitat patches. Mechanistic movement models typically represent butterfly movement as a series of steps and turns which are used in a correlated random walk to simulate the flight path of a butterfly over time [57–59]. Our model is conceptually similar to a recent approach in which movement over time is represented as transitions between flights and inter-flight periods [10], rather than as fixed flight times for all butterflies. This allows representation of the changing durations of flights and inter-flights with environmental conditions and between the sexes (Fig. 1) and thus allows prediction of movement rates across a range of weather conditions. Durations of flight and inter-flight periods are drawn from solar-intensity and temperature-specific log-normal distributions with the parameters predicted through model fits to the observed changes in parameters across weather conditions (*described above*). Individuals in the model move during a flight by random draws from observed distributions of step lengths and turning angles. An overview of the model is given in Fig. 1. Each individual first selects an inter-flight duration and remains stationary until this time has elapsed, and then it draws a flight duration. To move during flight the individuals draw step distances from marginal distributions of step lengths observed for flights of that duration. For example, if a four second flight was drawn a corresponding step from the four second marginal distribution of step lengths would be selected. The butterfly then moves forward at a rate such that the step length is completed in the flight time. As step lengths were measured at a maximum of every 15 s a long flight may result in multiple steps being drawn before the flight time has elapsed. This detail, which is not included in standard random walk approaches, decouples movement rate from flight time and is important here to fairly represent the effect of changing flight durations on movement*.* After a flight, or every 15 s during flight, the individuals change heading by drawing a turning angle and adding this turn to the current heading. After the flight time had elapsed the individuals selected another inter-flight duration, and this was repeated until the end of the simulation. To match field observations as closely as possible observations of butterflies ceased after 20 or 15 flags had been laid at the proportions used in field observations, and a low probability of being lost in flight was included. The model was built in NetLogo 6.0 [60] and analysis was carried out using the RNetLogo package [61]. Von-Mises circular distributions were fitted to observed turning angles using the ‘*circular*’ package in R [55, 62].

## Results

### Short term movements of individual butterflies

**Fig. 2:**
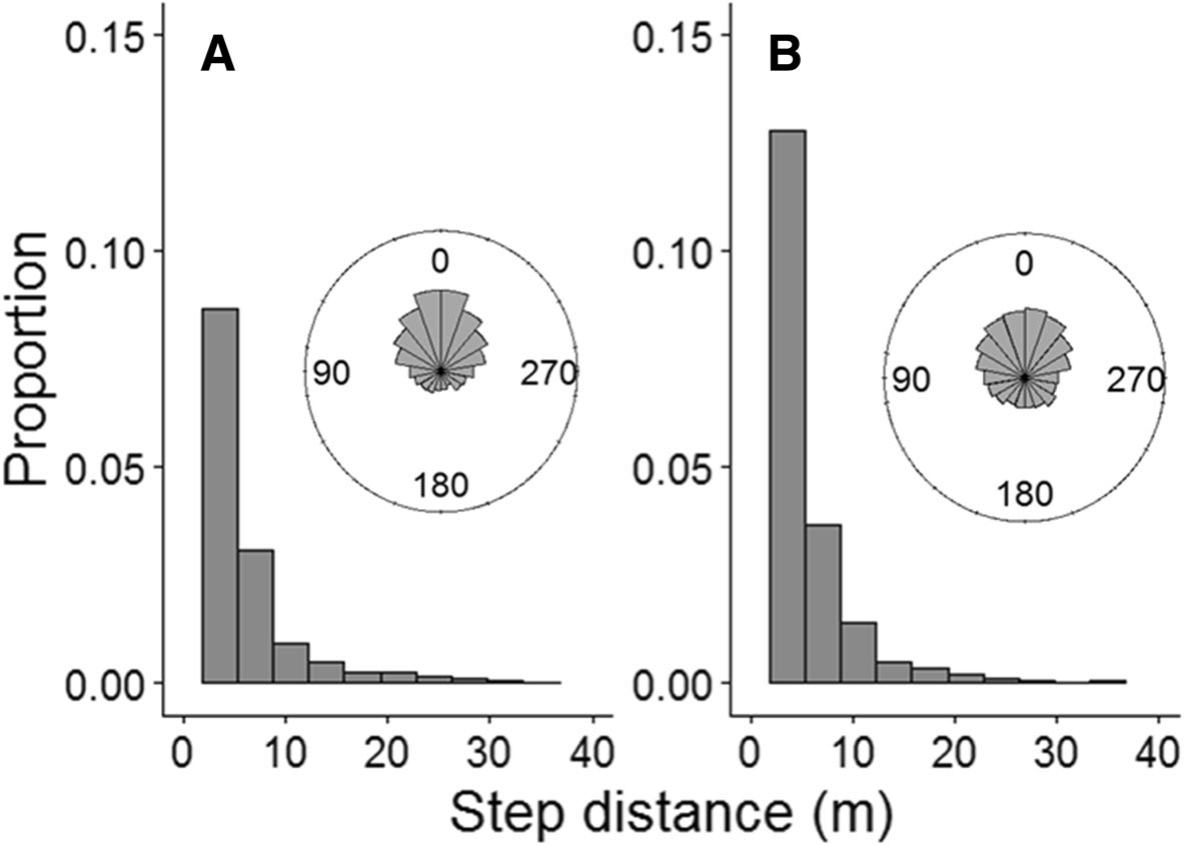
Step distances with relative turning angle inset for **a**) males; **b**) females

The positions of individual butterflies were marked when they took off, when they landed, and every 15 s during flight: the distance between successive marks is referred to as a step, and the change in direction between successive steps is referred to as a turn. Males had significantly longer step distances than females (mean ± SE: females = 3.21 m ± 0.16 m; males =3.88 m ± 0.11 m*,* t-test on log step distances: *t* = 5.09, *p* < 0.001, df = 1351.1) and more directed flights (circular mean resultant length: females = 0.40, males = 0.61, Wallraff test: X^2^ = 34.4, *p* > 0.001) (Fig. 2) but females flew faster than males as measured by step speeds (step distance/step duration) (Table 1). Step speeds were not influenced by solar radiation and there was only weak evidence of an effect of air temperature or (air temperature)^2^ though they were both retained in AIC model selection (Table 1).

**Table 1:** Effects of sex and environmental variables on flight and movement characteristics

	Step speed (m/s)	Inter-flight duration (s)	Flight duration (s)	Displacement rate (m/s)	Distance rate (m/s)
Sex (M)	−0.13*** (± 0.03)	− 1.1*** (± 0.14)	1.19*** (±0.11)	0.11*** (±0.02)	0.175*** (±0.03)
Air temperature (°C)	−0.06• (± 0.03)	0.27• (± 0.16)	0.55*** (±0.13)	0.07** (±0.02)	0.064* (±0.02)
Air temperature^2^ (°C)	0.001• (± 7 × 10^−4^)	−0.006 (± 3.6 × 10^−3^)	−0.02*** (±2.8 × 10^− 3^)	− 0.002** (±4.7 × 10^− 4^)	−0.001* (±5.7 × 10^− 4^)
Solar radiation (Lux)	–	− 1.04 × 10^− 5^*** (± 1.26 × 10^− 6^)	1.31 × 10^− 6^ (±8.99 × 10^− 7^)	7.28 × 10^− 7^*** (1.7 × 10^− 7^)	1.35 × 10^− 6^*** (1.9 × 10^− 7^)
DF	142	276	211	233	266
R^2^	0.17	0.36	0.39	0.24	0.33

Analyses performed using linear models, predictors removed or retained through AIC model selection. • < 0.1, * *p* < 0.05; ** *p* < 0.01; *** *p* < 0.001. Numbers in parentheses indicate standard error of the estimated coefficients

### Behaviour over 10 min

**Fig. 3:**
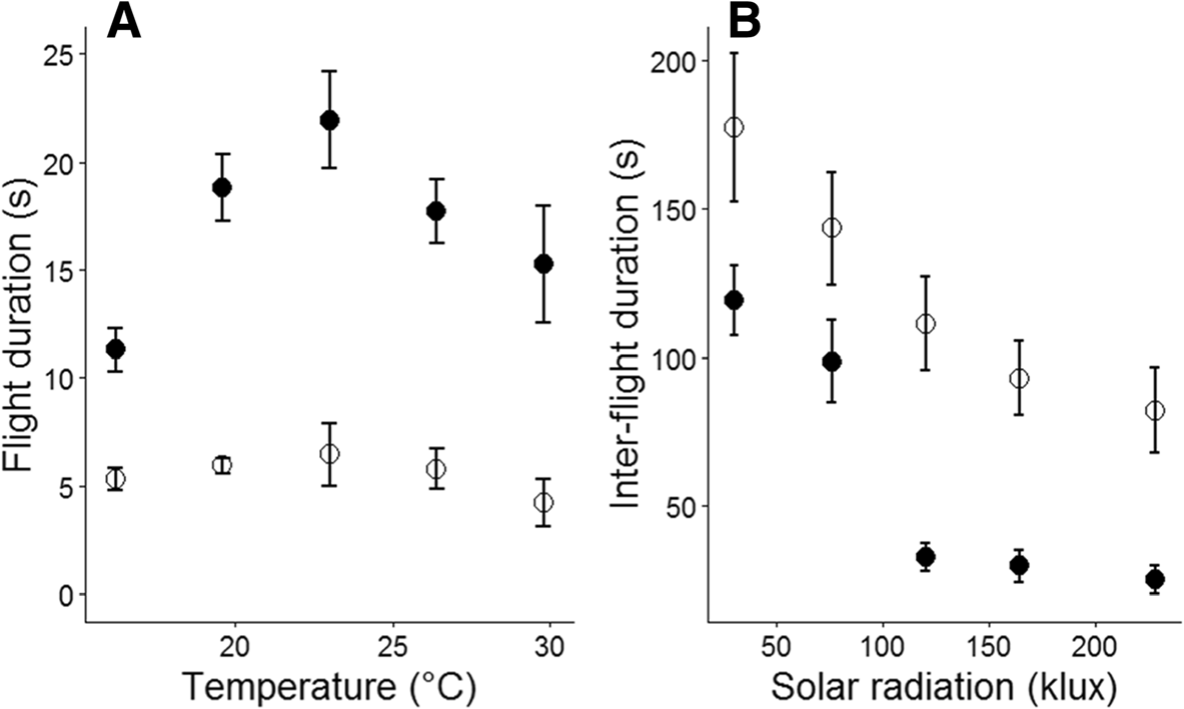
**a**) Flight durations across temperature categories; **b**) inter-flight durations across solar-radiation categories. Male butterflies shown as solid circles, females as open circles

Males were significantly more active than females, with longer flights (Fig. 3a, median flight durations: males: 9.1 s, females 3.8 s) and shorter inter-flight durations (Fig. 3b, median inter-flight durations: males 15.1 s, females 38.8 s) (Table 1). In addition to the effects of sex, flight durations were affected by air temperature but not solar radiation, while inter-flight durations were most affected by sex and solar radiation, with weak evidence for an effect of air temperature (Table 1). Flight durations increased with air temperature and peaked between 20 °C and 26 °C, and then decreased, but only marginally so for females (Fig. 3a). Inter-flight durations declined as solar radiation levels increased (Fig. 3b). Males had higher displacements rates than females (Table 1). For displacement and distance rates, which integrate effects on flights and inter-flight durations, air temperature, (air temperature)^2^ and solar radiation all significantly affected observed rates.

### Generalising behaviour with log normal distributions

Quadratic models fitted to the parameters of log-normal distributions (log μ, σ) were used to generalise the non-linear behavioural changes of *M. jurtina* across weather conditions (coefficients presented in supplementary materials 1). The effect of insolation on inter-flight durations was well captured using this approach fitting closely the parameters of the log-normal for both sexes (R^2^: Males log μ = 0.94, σ =0.91; Females log μ = 0.98, σ =0.88). For male butterflies parameters of flight durations across air temperatures, were also well fitted (R^2^: log μ = 0.86, σ =0.81) though for females the effect of air temperature was generally much weaker (Fig. 3a) and with no simple relationship between the log-normal parameters and air temperatures a data driven approach was applied by using the fitted parameters for an air temperature category when simulating air temperatures within that interval in the individual-based model.

### Using the individual-based model to predict dispersal rates

The individual-based model described in Methods was developed to bridge the gap between short-term observations of movements and 10-min displacements by explicitly representing changes in behaviour across weather conditions. The model uses weather-dependent parameterisations (supplementary material 1) of flight durations and inter-flight durations to predict movement rates, measured as distance rate (track path length/observation time) (Fig. 1) and displacement rates (Euclidean distance/observation time) (Additional file 1: Figure S2).

**Fig. 4:**
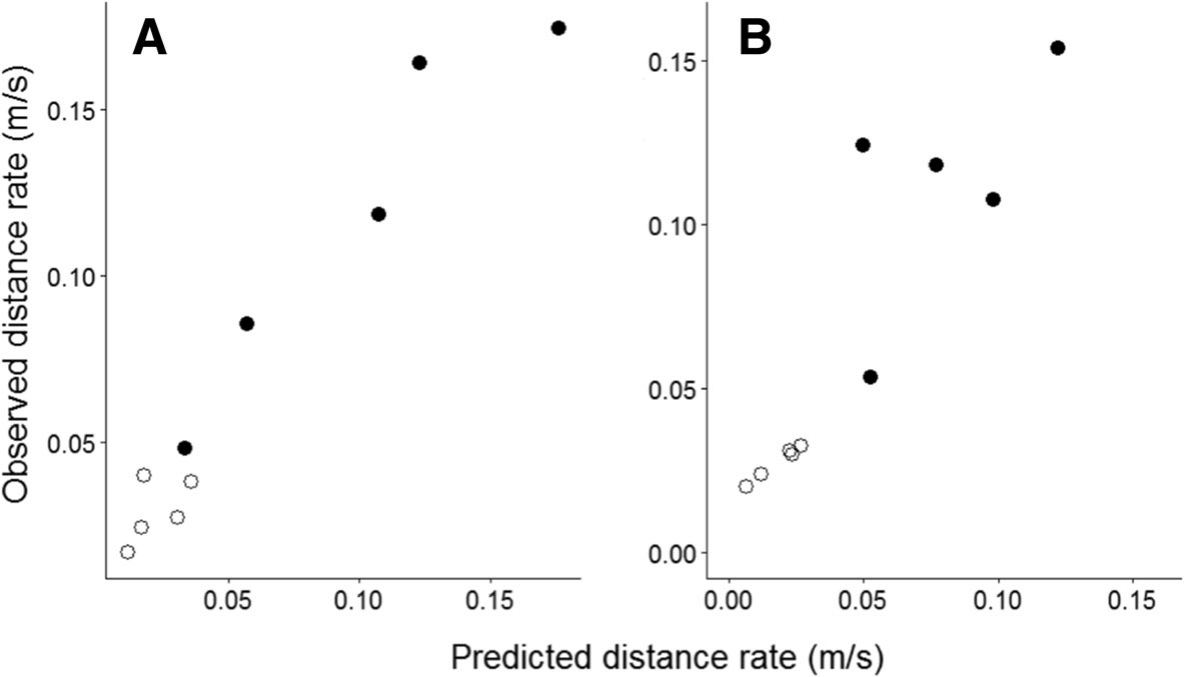
Comparison of model predictions with observations of distance rate for males and females **a**) sunshine categories and **b**) Temperature categories. Male butterflies shown as solid circles, females as open circles

The model was validated by comparing predictions of movement rate with the observations for each air temperature and solar-intensity level (Figs. 4 and Additional file 1: Figure S2). Predictions were obtained by inputting the air temperature and solar radiation of a field observation, running the model for ten minutes of simulated time and then collecting the measure of displacement, this process was repeated 20 times per individual. Distance rates are preferable for validation because they are not sensitive to edge-of-habitat effects, which are not included in the model, but displacement is a more direct measure of 10-min displacement because it represents the Euclidean distance moved.

Predicted and observed distance rates were highly correlated across of levels of sunshine (Fig. 4a, Pearson’s *r* = 0.97, *p* < 0.001) and air temperatures categories (Fig. 4b, *r* = 0.90, *p* < 0.001) though there is some under-prediction for males at the two highest temperature categories. Similarly high correlations were obtained for displacement rates across sunshine categories (Additional file 1: Figure S2A, Pearson’s *r* = 0.89, *p* < 0.001) and temperature categories (Additional file 1: Figure S2B, Pearson’s *r* = 0.90, *p* < 0.001). We consider that these high correlations between observations and predictions constitute satisfactory validation of the model.

**Fig. 5:**
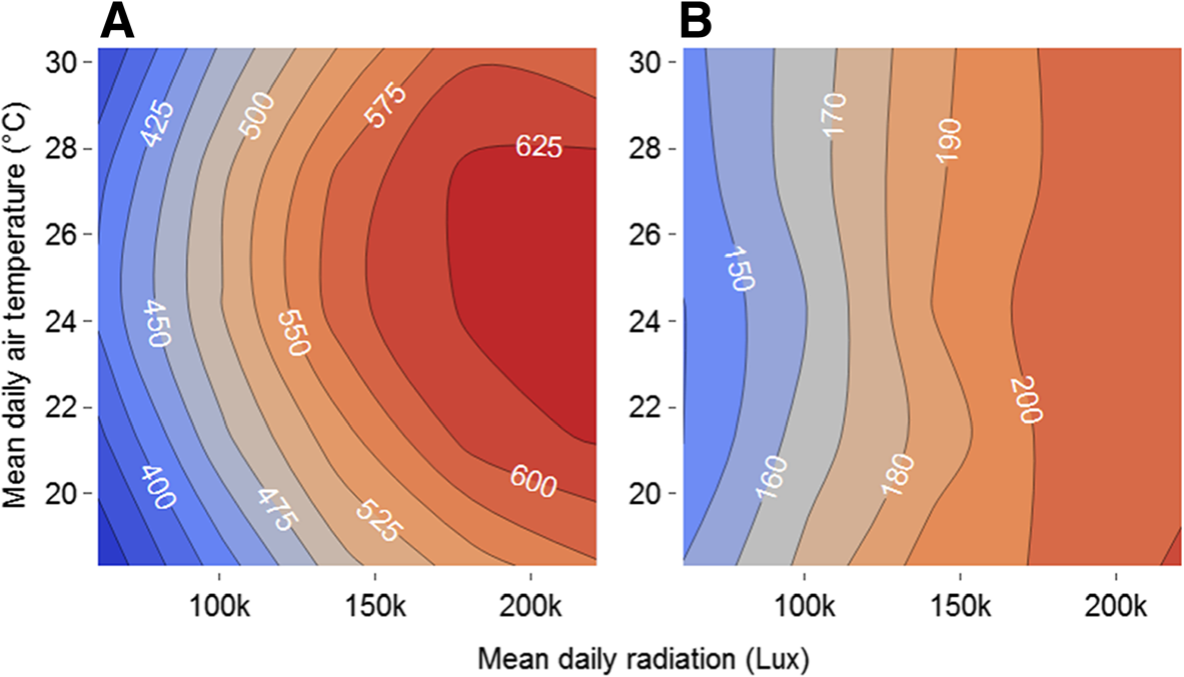
Predicted mean weekly displacements (m) for a given combination of solar radiation and air temperature for **a**) Males and **b**) Females

To analyse the effects of solar radiation and temperature on movement over a meaningful timeframe for the dispersal potential of a population, simulations of the movement of 1000 butterflies over a week (5 days × 8 h) were performed for 25 simulated weather conditions (5 sunshine × 5 temperature levels). Daily temperatures were simulated by fitting a Loess curve to observed temperatures during the 2018 field observations and shifting the intercept of the function in 3 °C intervals to replicate cooler or warmer days (Additional file 1: Figure S1). Daily sunshine levels were similarly replicated by fitting a custom function to observed solar radiation and shifting the intercept in 20 klux intervals (Additional file 1: Supplementary materials 2). Weather changes occurred half-hourly in the simulation and on-going behaviours, such as inter-flight durations, then ceased and a new behaviour was drawn, so that butterflies were reactive to the changing conditions. Maximum mean weekly displacements were predicted approximately three times greater for males than for females (Fig. 5). The range of weekly displacement predictions varied more than two-fold across solar-intensity and temperature categories for males and > 50% for females. For both sexes predicted weekly displacement responded strongly to solar radiation. Displacement peaked at intermediate temperatures in males, but there was no strong effect in females. These results were similar for distances travelled (Additional file 1: Figure S3) with males flying much further than females and flying furthest at intermediate temperatures, and both sexes travelling further distances with increasing solar-intensity.

## Discussion

Our objective has been to integrate the effects of air temperature and solar radiation into an individual-based model which predicts movement rates for *M. jurtina*. Our method has been to identify the short-term effects of the weather variables on flight and inter-flight durations (Fig. 3 and Table 1), and then to draw from distributions representing these weather-dependent behaviours within the individual-based model. Two measures of movement are presented: displacement rates and distance rates, and the model is satisfactorily validated for both measures by comparing observations and predictions (Figs. 4 and Additional file 1: Figure S2). The model is subsequently used to analyse the effects of weather on weekly displacement and demonstrates that within the analysed range weather has a greater than two-fold effect for males and greater > 50% for females (Fig. 5).

Weather strongly influences butterfly behaviour, primarily through the effects of air temperature on flight duration, and solar radiation reducing the time interval between successive flights (Fig. 3). These effects of weather on movement are consistent with theoretical expectations based on biophysical analysis and observations of thermoregulatory behaviour [63–66] and consistent with previous observations of butterfly movement [20, 23, 29, 67]. While warmer temperatures are predicted to increase the scope for muscle power by enhancing aerobic capacity [68], we found no strong evidence of a relationship between flight speed and either air temperature or solar radiation. It is likely that the flight speed measured in this study reflects a foraging strategy optimised for favourable habitats rather than a maximal rate [69]. Therefore, a limitation when relating our results to longer-term dispersal is the complexity of the dispersal process with movement behaviour changing between habitat types [51] and influenced by edge effects [70]. Nonetheless, the influence of weather on behaviour was found to account for more than a two-fold variation in displacement rate, which is consistent with observed annual variability in dispersal rates [28].

While both sexes showed similar flight speeds, males had longer flight durations and shorter intervals between successive flights, resulting in a three-fold greater predicted daily displacement. These sex differences likely reflect different priorities. Male *M. jurtina* continuously ‘patrol’ habitat in search of females to mate with, whereas mated females search for suitable host plants on which to lay eggs [20, 45]. While males appear to maximise flight durations on sunny days when solar radiation can be used to elevate body temperature, females show reduced activity which is less temperature dependent. This restricted flight period for oviposition may ultimately reflect thermal constraints on egg maturation rate [71]. The optimal strategy for females may be to fly only when eggs are ready to lay, to minimise unwanted attention from males and associated energetic costs.

Although below 23 °C temperature had a positive effect on flight duration, for male butterflies flight durations declined above 26 °C (Fig. 3). Similarly, predicted displacement for males peaked at approximately 26 °C, and afterwards declined, though there was no strong effect of temperature on females (Fig. 5). For both sexes movement predictions peaked at the highest solar radiation levels. Declines in activity and switches in behaviour are consistent with ectotherms nearing their thermal limits [40] . High temperatures have for instance been shown to reduce mate-searching behaviour in the small white (*Pieris rapae*) [72]. Our results suggest that while a warmer climate is likely to increase potential dispersal rate and potentially population stability for *M. jurtina* [29], particularly at its northern range boundary, predicted high temperatures under climate change might ultimately restrict movement with detrimental effects on the stability of populations unless accompanied by an associated change in phenology, population size, habitat use and/or thermal adaptation [73, 74], such as seen in the morphological differences in species of Colias butterflies across altitudinal gradients [23].

While the long-term ecological consequences are complex to predict, we have demonstrated that the current relationship between behaviour and weather can be defined and included in mechanistic movement models. The temperature-dependence of flight behaviour observed particularly for male *M. jurtina,* has a number of important general implications. Firstly, weather alone may explain much of the variation in movement observed for butterflies among sites and among years [28, 31], and therefore ought to be accounted for when estimating butterfly and other ectotherm movement behaviours. Secondly, the influence of weather on dispersal may affect population synchrony in both space and time [75]— the Moran effect [76]. Thirdly, the finding that flight behaviour is constrained by unfavourably hot conditions suggests opportunities for oviposition may be more limited than previously thought, reducing the possible benefits of temperature dependent increases in fecundity [77].

We hope that the approach of representing the weather dependence of movement in models can be applied more generally across species, using mechanistic understanding of how movement depends on traits differing between species such as body size [64, 78], thermoregulatory behaviour and melanism [25, 65], or observation of thermal performance curves on a species by species basis. Thermal performance curves for movement are available for several insects [79–81], and reptiles [82–84]. We hope that in this way the effects of changing climate may be better predicted using mechanistic movement models that account for the effects of varying environmental conditions.

## Conclusions

Individual based models provide a useful framework for including mechanism in movement models. By disentangling the effects of weather on different aspects of flight behaviour, and then by demonstrating how to integrate these insights into an individual based model of butterfly movement, we were able to explain up to a two-fold difference in movement rate of *M. jurtina* consistent with inter-annual variation in dispersal measured in population studies. We have also revealed that climate change for the studied populations, may be expected to decrease activity and dispersal rates since these butterflies already operate close to their thermal optimum. We hope that developments of our model will enable improved forecasting of the ecological consequences of changes in weather, and ultimately climate, and provide impetus to include greater mechanism in future movement models.

## Additional file

Additional file 1: Supplementary materials 1, 2, 3 and 4. (DOCX 3035 kb)

## Data Availability

Should the manuscript be accepted, the data supporting the results and all model code, will be archived in a public repository such as Dryad or Figshare and the data DOI will be included at the end of the article.
