# Supplementary material for: Integrating the influence of weather into mechanistic models of butterfly movement
Source: Mov Ecol. 2019 Sep 2;7:24. doi: 10.1186/s40462-019-0171-7 (PMC6717957; doi:10.1186/s40462-019-0171-7)
Supplement: Supplementary file 1 — Supplementary materials 1, 2, 3 and 4. (DOCX 3035 kb) [file 40462_2019_171_MOESM1_ESM.docx]

**Supplementary material 1**

Results of quadratic model fits for parameters of log normal distributions representing changing flight and inter-flight durations with weather conditions.

*Males:*

Flight durations

Log μ = -2.365 + 0.397 x Air temperature (°C) - 0.008 x (Air temperature)² (°C)

Log σ = 0.120 + 0.085 x Air temperature (°C) - 0.002 x (Air temperature)² (°C)

Inter-flight durations

Log μ = 4.261 – 1.589 x 10⁻⁵ x Solar radiation (Lux) + 3.129 x 10⁻¹¹ x (Solar radiation)² (Lux)

Log σ = 2.561 – 1.289 x 10⁻⁵ x Solar radiation (Lux) + 3.415 x 10⁻¹¹ x (Solar radiation)² (Lux)

*Females:*

For female butterflies there was no clear response between the parameters of the log-normal and changing air temperatures so instead the parameters fitted across temperature intervals are provided.

| Air temperature (°C) | Log μ | Log σ |
| --- | --- | --- |
| > 14.5 -- < 17.9 | 1.377 | 0.825 |
| > 17.9 -- < 21.3 | 1.404 | 0.911 |
| > 21.3 -- < 24.7 | 1.204 | 0.984 |
| > 24.7 -- < 28.1 | 1.175 | 1.043 |
| > 28.1 | 0.705 | 1.353 |

Inter-flight durations

Log μ = 4.763 – 9.495 x 10⁻⁶ x Solar radiation (Lux) + 1.880 x 10⁻¹¹ x (Solar radiation)² (Lux)

Log σ = 2.538 – 9.185 x 10⁻⁶ x Solar radiation (Lux) + 2.371 x 10⁻¹¹ x (Solar radiation)² (Lux)

**Supplementary material 2**

To generate realistic changes in daily temperature and solar radiation, two approaches were used. For air temperature, a Loess curve was fitted to air temperature observations from the on-site meteorological station at University of Reading throughout the days of the field observations of 2018 (Fig. 6). Observations were recorded every 5 minutes. The curve was used to predict a standard function of air temperatures between 9:00 to 17:00 and changes in air temperature between days were modelled by increments of ± 3°C to the standard curve. For solar radiation the dataloggers used measured a broad spectrum of light wavelengths and they are most effective at measuring the relative light intensity, though through concurrent observations of light intensity from the University of Reading meteorological station measurements could be related to the spectrum of light intensity commonly reported. The conversion of lux between the meteorological station and the dataloggers was approximated by doubling the lux recorded at the meteorological station. This produced upper and lower bounds of solar radiation that matched well those recorded using the dataloggers. Loess curves were ineffective at matching the observed shape of solar radiation through time, so a custom exponential function was used to model the shape of the solar radiation with difference in days controlled by increments of ± 20000 lux to the standard curve.


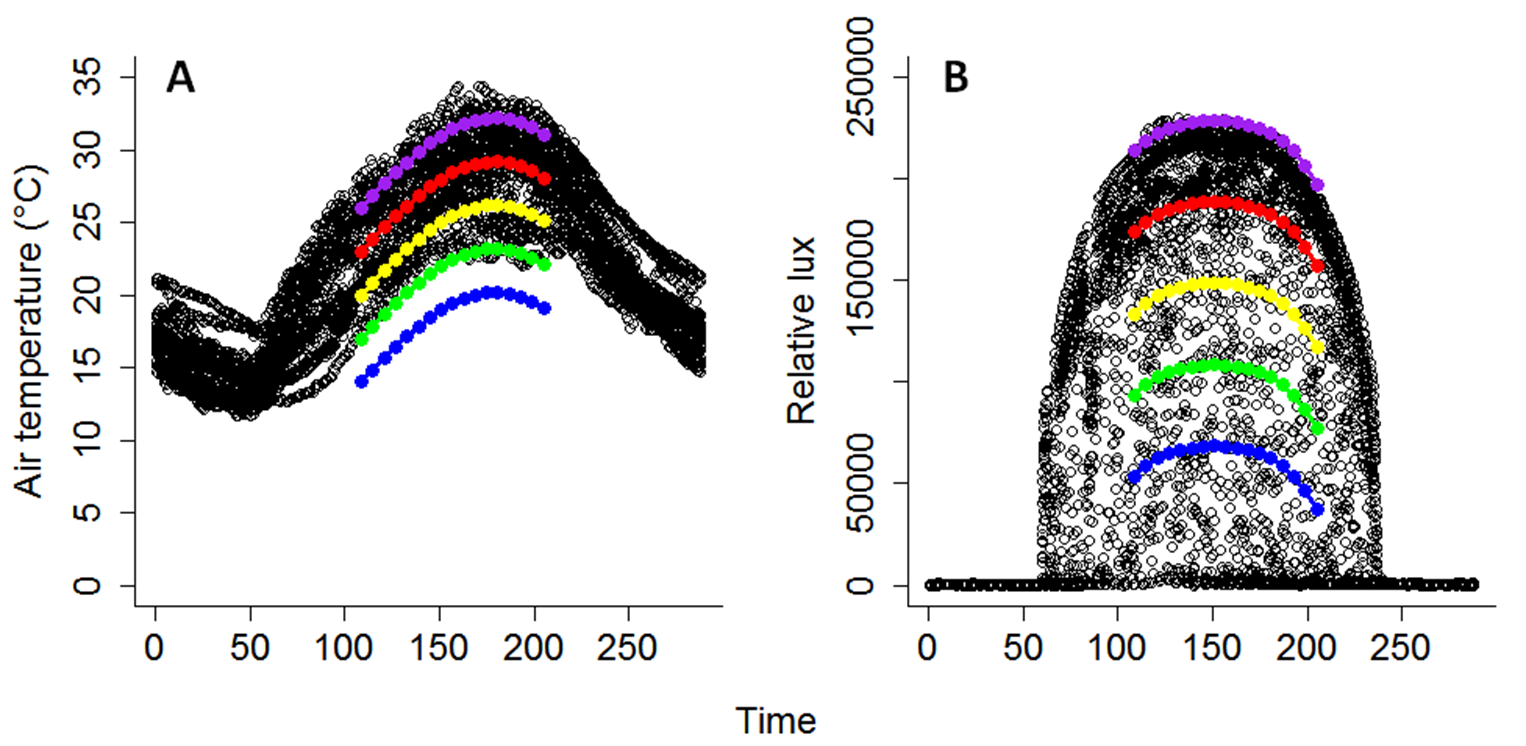
Figure 6. Observed and modelled weather observations. A) Air temperature, B) Solar radiation. Black points show observations and coloured points and lines simulations across weather conditions.

**Supplementary materials 3**


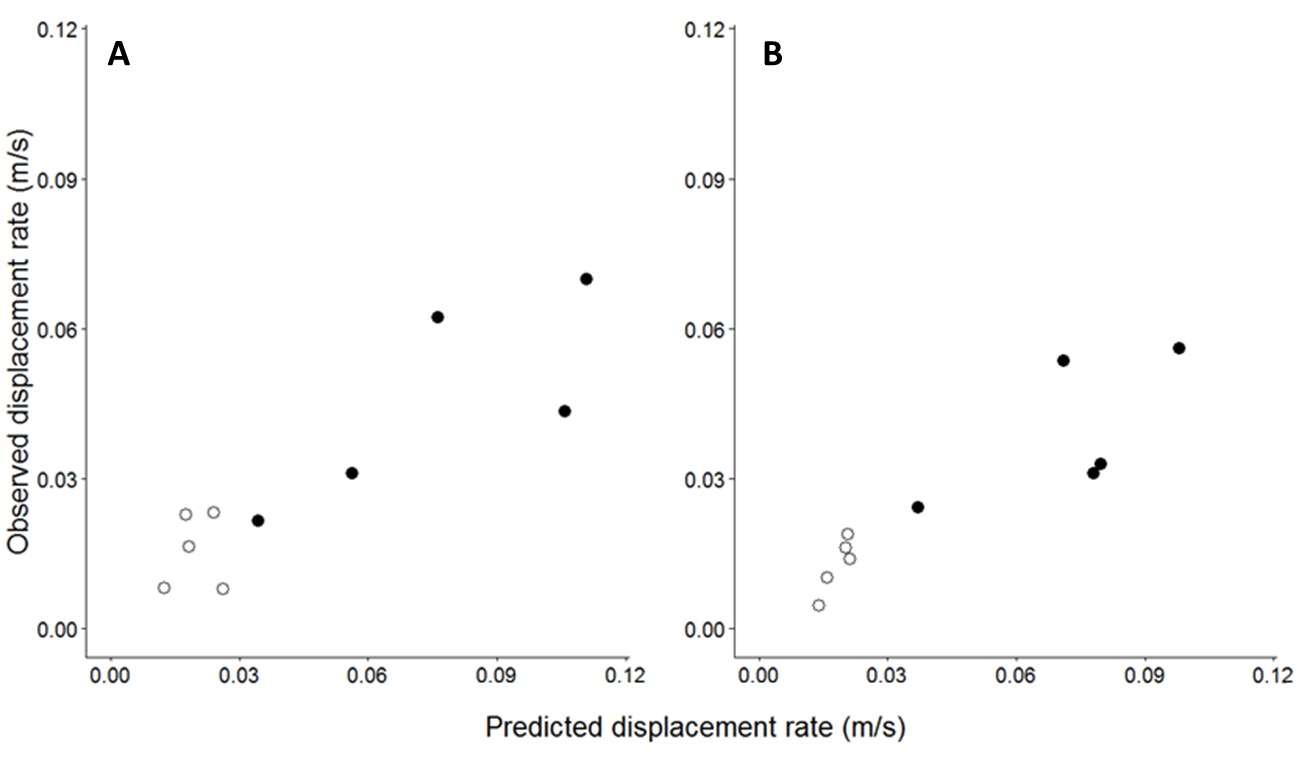


Figure S2. Comparison of model predictions with observations of displacement rate for males and females A) sunshine categories and B) Temperature categories. Male butterflies shown as solid circles, females as open circles.

**Supplementary materials 4**

**
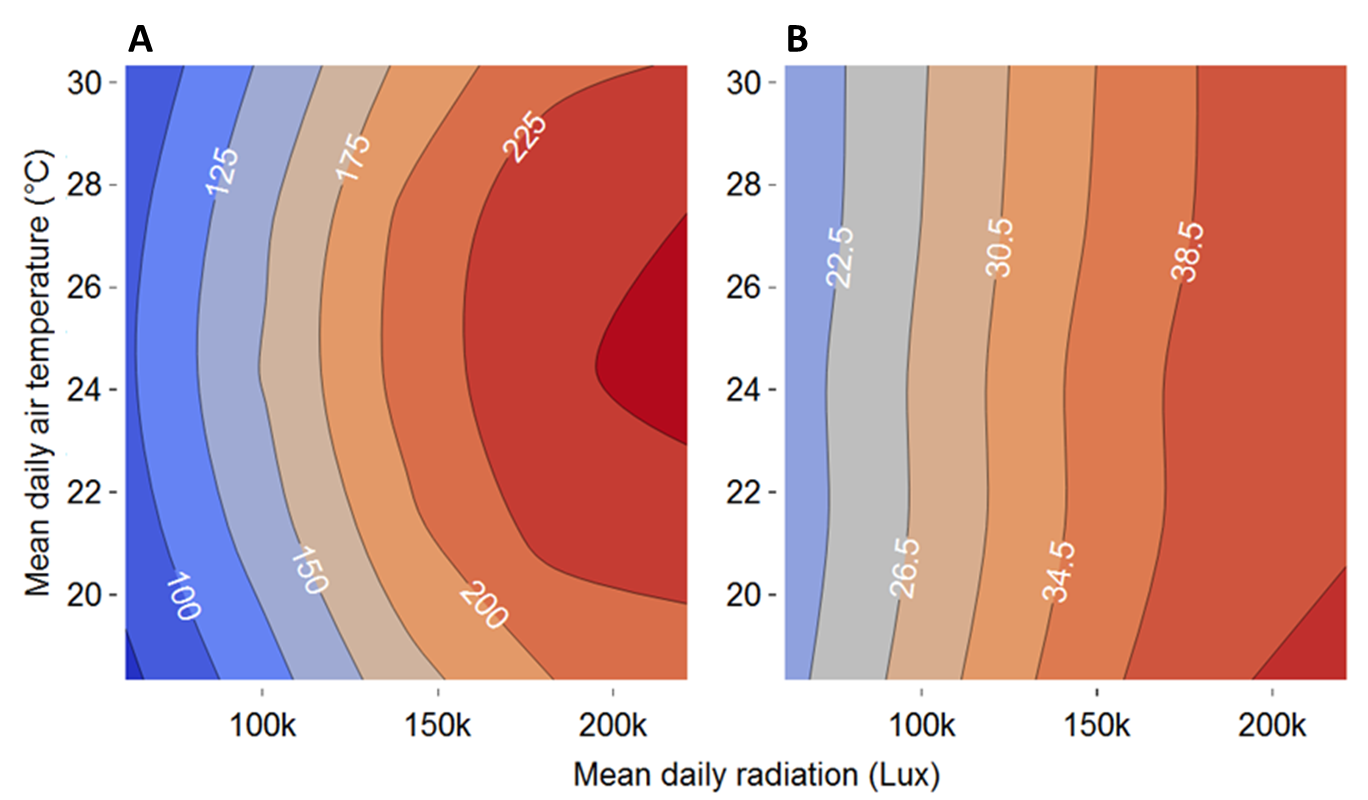
**

**Figure S3**. Predicted weekly distances x 10² (m) for a given combination of solar radiation and air temperature for A) Males and B) Females.

**Supplementary materials 5**

**
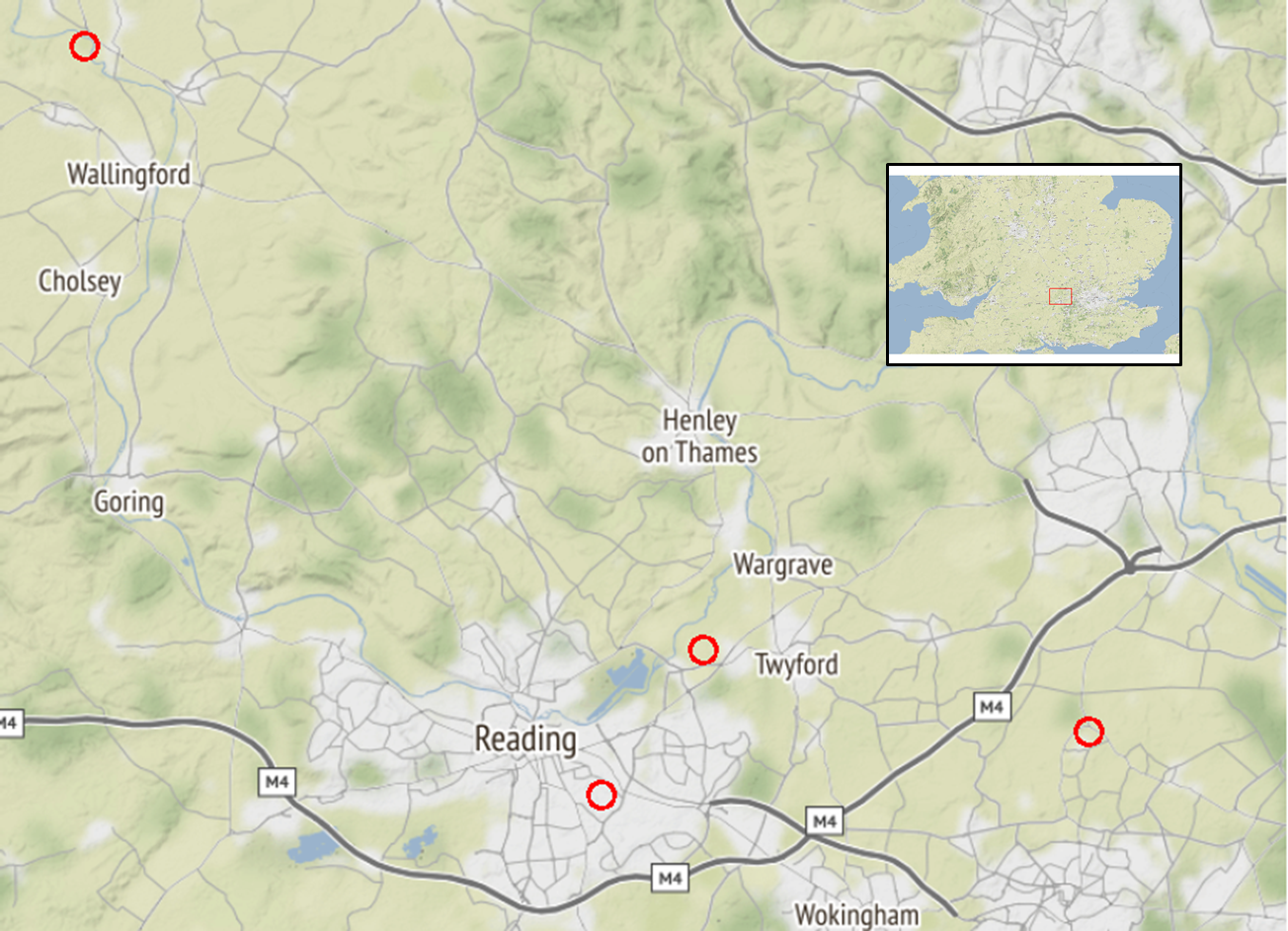
**

Figure S4. Map of study sites. Location within Southern England inset.
